# Polypus of the Œsophagus

**Published:** 1871-08

**Authors:** J. T. Everett

**Affiliations:** Sterling, Illinois


// CHICAGO
### MEDICAL EXAMINER:

N. S. DAVIS, M.D., EDITOR.

F. H. DAVIS, M.D., ASSISTANT EDITOR.

VOL. XII. AUGUST, 1871. NO. 8.

### Original Contributions.

### POLYPUS OF THE ŒSOPHAGUS.

BY J. T. EVERETT, A.M., M.D., Sterling, Illinois.

Mrs. G., Irish, aged 40, married, mother of one child, who is now 18 years of age. Was called to see Mrs. G. April 5th, 1871, at 2.30 P.M. Elicited the following previous history of her case:

Patient had been troubled for five years with a sense of some foreign substance in the throat. Had been treated for cancer, consumption, tapeworm, etc., by both quack and regular. Her general health had been gradually failing. Was taken about a week before I called with severe dyspepsia, nausea, apyrexia, cephalalgia, and cough. Found patient in low typhoid condition, extremities cold, radial pulse, at times imperceptible; circulation weak and deficient. Cerebral functions impaired; bowels had not moved for five days; had slept none for seven days (so the friends affirmed); respiration short and labored, 24 per minute; pulse 115; temperature of axilla, 98·5°. After a careful study of the case I could not agree with the previous diagnoses. There was not the cachectic appearance of carcinoma, neither had there been the sharp pain felt in cancer. There were none of the symptoms of tapeworm, and the patient had had poor appetite for years. Percussion and auscultation showed both lungs quite sound, and an entire absence of signs indicating the other troubles, led me to diagnose the case as above.

From the typhoid condition of the patient there was evidently some morbid principle in the blood, but from what source? The patient, it is true, lived in rather an unhealthy locality, but the previous history of the case showed it was not simple typhoid fever. I therefore reasoned that from want of nutrition, or some other occult cause, the polypus was suffering softening and disintegration, and that the decaying matter passing down into the stomach, and being absorbed into the circulation, was producing its noxious effects upon the system. This theory and the diagnosis of the case was rendered certain by the subsequent ejection from the stomach of pus, shreds of fibrous tissue, and a large portion of the polypus entire.

The indications then for treatment were: To re-establish circulation, to allay vomiting, to procure sleep, to stop the cough, and to move the bowels. To effect the first indication I ordered:

℞. Tinct. veratrum viride................ʒß.

Tinct. nux vomica ....................ʒj.

Syrup simp. ..........................℥ij.

M.S. Teaspoonful once in four hours.

To allay the vomiting I ordered:

℞. Tinct. ferri chloride .................ʒij.

Potassa chlorate ......................ʒij.

Carbolic acid..........................6 grs.

Syrup simple ..........................℥iij.

M.S. Teaspoonful once in four hours, alternating with No. 1.

*April 6th, 10 A.M.* Patient conscious; feet and hands warm; pulse soft, 100 per minute; temperature, 99.5°; respiration, 18; skin dry and hot; no appetite. Patient has had no rest; no movement of bowels; vomiting less frequently. Ordered:

℞. Tr. Ferri chloride ............... ʒij.
Quinia sulph. ..................... grs. 20.
Strychnia .......................... grs. j.
Syrup simple ....................... ℥iij.

Sig. in place of No. 1. To procure sleep, gave chloral, grs. 10, every hour for six hours.

*5.30 P.M.* Vomiting nearly ceased; patient easier. To allay cough, ordered:

℞. Ammonia chloride ................. ʒij.
Morphia sulphate ................... grs. iij.
Anti. et potass. tart. ............. „ ij.
Syrup glycyrrhiza .................. ℥iij.

M. S. Teaspoonful once in four hours. Ordered chloral grs. 30.

*April 7th, 10 A.M.* Vomiting less frequently; cough less troublesome; slept well. Nurse showed me a piece of fleshy substance which patient had vomited, which examination proved to be the offending polypus.

*April 7th, 4.30 P.M.* Patient feels easier; has not vomited since morning. Cough much abated; respiration normal; pulse 60. Ordered:

R. Quinia sulph. ..................... grs. 30.
Strychnia .......................... „ j.
Aqua camph. ........................ ℥j.
Syrup simp. ........................ ℥ij.

M. S. Teaspoonful three times daily.

*April 8th, 10 A.M.* Found patient unconscious, apparently in state of collapse; muscles relaxed; cold perspiration over whole body; no pain; no vomiting; no cough. Nurse said last medicine seemed to distress patient. Called for bottle to see what was the matter. Found a white floculent matter in a milky-looking fluid; concluded there was too much camphor. Ordered ammonia carbonate, grs. ij., once in twenty minutes, until reaction took place. Took bottle to office, and on analyzing, found that each teaspoonful contained about 6 grs. gum camphor.

*12.30 P.M.* Patient conscious, but hardly rallied. Continued ammonia carbonate.

*April 8th.* Went to drug store, and found that clerk had used spts. camph. instead of aqua.

*April 9th, 10 A.M.* Patient fully rallied, but weak and faint; rested well; skin moist; pulse 70; respiration normal; temperature 100°. Injection this morning produced free evacuation.

*5.30 P.M.* Patient rested well; took beef tea; vomiting entirely ceased.

*April 10th.* Patient chilly, with sense of gastric oppression. Begged hard for an emetic; gave syrup ipecac, ʒj. Free emesis of large quantity of ropy mucus, with fibrous shreds and pieces of fleshy substance.

From this time on patient made rapid recovery, and called at office to-day to pay bill (May 15th), and say she was well.

